# Mechanical Behavior of PEEK and PMMA Graphene and Ti6Al4V Implant-Supported Frameworks: In Silico Study

**DOI:** 10.3390/ma18020441

**Published:** 2025-01-18

**Authors:** Mariano Herrero-Climent, Fernando Sanchez-Lasheras, Jordi Martinez-Lopez, Javier Gil, Aritza Brizuela-Velasco

**Affiliations:** 1 Porto Dental Institute, Av. De Montevideo 810, 4150-518 Porto, Portugal; dr.herrero@herrerocliment.com; 2 University Institute of Space Sciences and Technologies of Asturias (ICTEA), University of Oviedo, 33004 Oviedo, Spain; fsanchez@tecniproject.com; 3 Department of Mathematics, Faculty of Sciences, University of Oviedo, 33007 Oviedo, Spain; 4 Soadco Research and Development Department, AD700 Escaldes, Andorra; j.martinez@soadco.com; 5 Biomimetics Oral Biomaterials and Interfaces (BOBI), Department Ciencia e Ingeniería de Matariales, Escola d’Enginyeria Barcelona Est, Universitat Politècnica de Catalunya, c/Eduard Maristany 16, 08029 Barcelona, Spain; 6 DENS-ia Research Group, Faculty of Health Sciences, Miguel de Cervantes European University, C/del Padre Julio Chevalier 2, 47012 Valladolid, Spain

**Keywords:** PEEK, G-PMMA, Ti6Al4V, finite element simulation, implant-supported frameworks

## Abstract

A comparative analysis has been carried out between three different dental materials suitable for the prostheses manufacturing. The analysis performed is based on the finite elements method (FEM) and was made to evaluate their performance under three different loading conditions. Three different materials were modeled with 3D CAD geometry, all of them suitable to be simulated by means of a linear elastic model. The materials employed were graphene polymethyl methacrylate (G-PMMA) with 0.25% of graphene, polyether ether ketone (PEEK), and Ti6Al4V. Three loading conditions have been defined: distal, medial, and central. In all cases under study, the load was applied progressively, 5 N by 5 N until a previously fixed threshold of 25 N was reached, which always ensures that work is carried out in the elastic zone. The behavior of G-PMMA and PEEK in the tests performed is similar. Regarding maximum deformations in the model, it has been found that deformations are higher in the G-PMMA models when compared to those made of PEEK. The highest values of maximum stress according to the von Mises criteria are achieved in models made of Ti6Al4V, followed by G-PMMA and PEEK. G-PMMA is more prone to plastic deformations compared to Ti6Al4V. However, due to its relatively higher stiffness compared to other common polymers, G-PMMA is able to withstand moderate stress levels before significant deformation occurs, placing it in the intermediate position between Ti6Al4V and PEEK in terms of stress capacity. It should be noted that there is also a difference in the results obtained depending on the applied load, whether distal, medial, or central, proving that, in all simulations, it is the distal test that offers the worst results in terms of presenting a higher value for both displacement and tension. The results obtained allow us to identify the advantages and limitations of each material in terms of structural strength, mechanical behavior, and adaptability to loading conditions that simulate realistic scenarios.

## 1. Introduction

When rehabilitating an edentulous arch, one of the possibilities that provides the best solution, both from a functional and esthetic point of view, is an implant-retained fixed prosthesis. However, the recommendation regarding the number of necessary support implants is diverse and ranges from recommendations of using one implant for each lost tooth [1,2,3], to only four to rehabilitate up to twelve teeth in occlusion, with optimal survival results, even in post-extraction and immediate loading protocols [4,5].

However, it is evident that the use of a smaller number of implants entails more challenging biomechanical situations, such as the tendency to increase the length of the pontics and favor deflection of the prosthetic structure or to use distal extensions, decreasing the mechanical advantage by increasing power arm. The choice of a correct prosthetic superstructure could possibly compensate for this mechanical behavior that tends to overload.

The prosthetic suprastructure is the part of the dental prostheses that is retained by the support implants, either directly through their connection or through an intermediate abutment, and, in the case of a full arch rehabilitation, can splint all the support implants. A dental prosthesis supported by several implants results in a combined structure in which the distribution of the applied loads depends on the relative rigidity of the various members involved, as well as the geometry of their distribution [6,7]. An osseointegrated implant forms an almost intimate connection with the bone, so it can be expected that the response to any load will be elastic, which means that the deflection of the fixation will be proportional to the applied load. On the other hand, with a rigid metal prosthesis, it can be assumed that its behavior will be relatively rigid compared to the implants, whose section diameter is smaller. The combined structure will normally be so complex that the static equations alone are insufficient to determine the distribution of the load performance, and the deformations of the prosthesis, implants, and jaw must be taken into account to determine said distribution. Such a structure is considered statically indeterminate and the analysis must treat the prosthesis in general and its suprastructure in particular as a curved elastic beam, subjected to bending and torsion [8].

In essence, this mechanical response of the suprastructures, when faced with the application of loads that come from masticatory forces, can lead to technical complications, of which fractures of these suprastructures are less frequent (0.2 to 4.7%) and the fracturing of the esthetic coating materials is more frequent (3.2% to 25.5%) [9].

However, as mentioned above, when evaluating the properties and mechanical behavior of a prosthetic suprastructure, it is too simple to do so in relation to its low tendency to fracture. These structures will have a greater or lesser capacity for deformation upon application of load and will consume a greater or lesser amount of tension in this deformation so that the rest of the system (retention screws, abutments, implants, and supporting bone) will receive more or less mechanical demand. In this regard, certain studies in the literature can be found that describe different degrees of deformation of the peri-implant bone depending on the material from which the prosthesis is made [10]. The deformation of the superstructure may also influence the technical complications of the covering materials, such as feldspathic ceramics, disilicates, and acrylic resins, with weak bonds to the frameworks, often due to mere mechanical entrapment, which, by having different elastic properties and different coefficients of thermal expansion, can favor failure.

In general, the most commonly used material in the manufacturing of these suprastructures has been metal, especially cobalt chrome alloys, using lost wax casting techniques [11,12]. However, the advent of CAD/CAM technology applications has led to a revolution in the possibility of using other different materials, most of them processed using milling or sintering-machining methods. Among these, titanium and its alloys stand out, such as grade V and zirconium oxide, as well as other polymeric materials that have advanced first as provisional rehabilitation materials but are now beginning to be used as definitive ones, although with reasonable doubts about its mechanical behavior [13,14,15]. Of the latter, Polyetheretherketone (PEEK) and polymethylmethacrylate (PMMA) stand out. PEKK biomaterials are polymeric elastic materials with good shock absorbance and fracture resistance and present ultra-high performance among all thermoplastic composites for excellent mechanical strength, chemical resistance, and high thermal stability (tensile strength 90–100 MPa, compressive strength similar and modulus of elasticity between 3.6 and 4.1 GPa) [16]. PMMA was initially used as a dental material in 1937, although it must be acknowledged that it has inferior mechanical properties (tensile strength 50–70 MPa, similar compression resistance, and modulus of elasticity between 2 and 3.1 GPa), when compared to PEEK [17,18,19,20]. In this sense, several strategies have been applied in order to increase the mechanical properties of PMMA polymer resins, almost all of which were based on the addition of inorganic fillers such as mesoporous silica nanoparticles (MSNs), titanium dioxide (TiO_2_), carbon nanotubes (CNTs), and also nano-sized graphene oxides (nGO) [21].

Despite everything, the mechanical properties of polymeric materials are very far from those of metals or ceramics. For example, Ti6Al4V has a tensile strength of 900–1200 MPa, a similar compressive strength, and an elastic modulus of 110 GPa [22,23,24,25,26,27]. Therefore, although the use of this type of polymeric materials opens new expectations in oral rehabilitation, due to its simplicity of manufacturing, price, ability to adhere to coating materials, adjustment possibilities, etc., it still raises doubts regarding its biomechanic behavior.

In principle, the trend in prosthetic materials is to try to avoid metallic materials because of the corrosion and ion release problems they cause [28,29,30]. For this reason, the materials usually used are titanium or titanium alloys, especially Ti6Al4V, due to their good mechanical properties and electrochemical similarity with the metals of dental implants, which are generally made of commercially pure titanium [31]. Ceramic materials are difficult to form and also have the problem of brittleness. One of the most tenacious materials is zirconia partially stabilized with yttria, which, due to phase transformations produced by tension, improves its tenacity. However, these improvements in mechanical properties are not sufficient for the mechanical bending loads that often occur in the oral cavity. These ceramic materials are nevertheless excellent with respect to dental esthetics [32,33,34]. That is why polymeric materials such as PMMA or PEEK are being tried as they are thermoplastic materials with good mechanical properties that will not have creep due to cross-linking of their structures or improvements in their mechanical properties. This improvement in response to masticatory loads can be achieved by introducing graphene, as is the case in this work. However, the amount of graphene that can be incorporated into PMMA is limited due to the black coloration that it acquires in the PMMA as the graphene content increases [35,36].

For this reason, the motivation for this in vitro experimental study arises: to use a finite element analysis that allows for the studying of the tensions and deformations of these materials subject to loading in a full arch rehabilitation design on four implants, with the objective of comparing its results with those of a high-performance material, such as Ti6Al4V, and, based on that, being able to infer its clinical applications.

## 2. Materials and Methods

**Figure 1:**
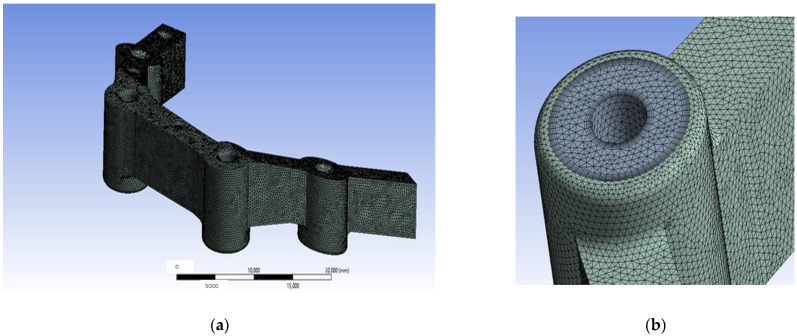
Finite elements model: (**a**) Meshed 3D view of the model employed for the present research; (**b**) detail of the mesh.

A 3D CAD model has been created. This model has an adaptive structured mesh in which the places where loads will be applied had especially been considered. As can be observed in Figure 1, this model has a total of 1,502,113 nodes that generate 1,024,036 elements, with an average quality of the element of 0.8116 [37], an aspect ratio [38] of 2.1113, and a Jacobian value of 1.0067 [39]. Also, its average deformation (skewness) is 0.21742 [40,41,42]. Figure 1 shows the 3D meshed model employed for the present research and details of the mesh. Although a formal mesh convergence test was not conducted, the results obtained for the mesh parameters presented before confirmed that the mesh met the criteria for numerical stability and accuracy.

Three different materials were modeled with 3D CAD geometry, as described before, all of which were suitable to be simulated by means of a linear elastic model. The materials employed were graphene polymethyl methacrylate (G-PMMA), polyether ether ketone (PEEK), and titanium alloy ASTM grade V (Ti6Al4V). Their main properties are listed in Table 1. The three models under study not only differ in the materials from which they are made of, but the models of G-PMMA and PEEK also have internal Ti6Al4V inserts in the area where they would be fixed to the other components. In the simulation, the prostheses are constrained with fully fixed supports at their base. In the referred models, the interfaces between pieces are defined as bonded, assuming perfect adhesion without relative motion, and under this assumption, no frictional contacts were required.

**Figure 2:**
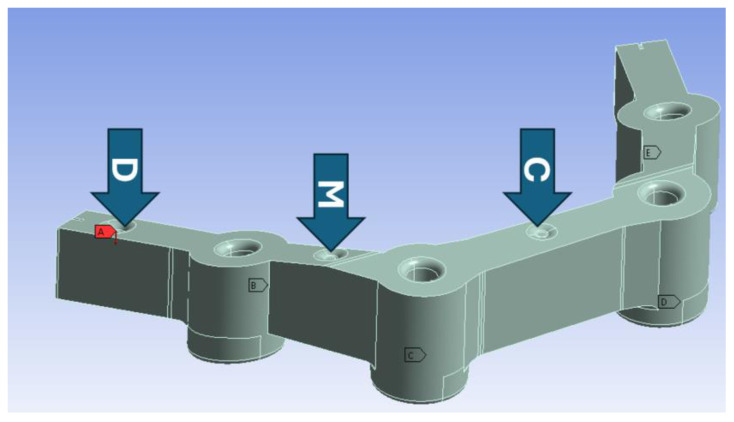
Load application points in the models under study (D: distal, M: medial, and C: central). Positions A, B, C, D and E are where dental implants are placed.

In this research, three loading conditions have been defined for each of the materials under consideration. The first one, with the model loaded onto one of its extremes, will be called distal; the one in which the load is applied between the first two inserts will be called medial; and, finally, the loading situation in which the load is in the center of the middle span will be called central. For these three load cases, control surfaces were generated. Please note that the generation of control surfaces is necessary because if the force were theoretically applied on a surface with a dimension of zero, the stress value would be infinite. Therefore, the use of control surfaces contributes to a more realistic model from a biomechanical point of view. Although each load is applied to a different model, Figure 2 represents the three loads applied to the same model for the reader be able to clearly see their location in the model and compare them.

In all cases under study, the load was applied progressively, 5 N by 5 N until a previously fixed threshold of 25 N was reached, which always ensures that work is carried out in the elastic zone for all materials. The value of 25 N was fixed considering that none of the materials would enter into the plasticity zone with such load value.

**Figure 3:**
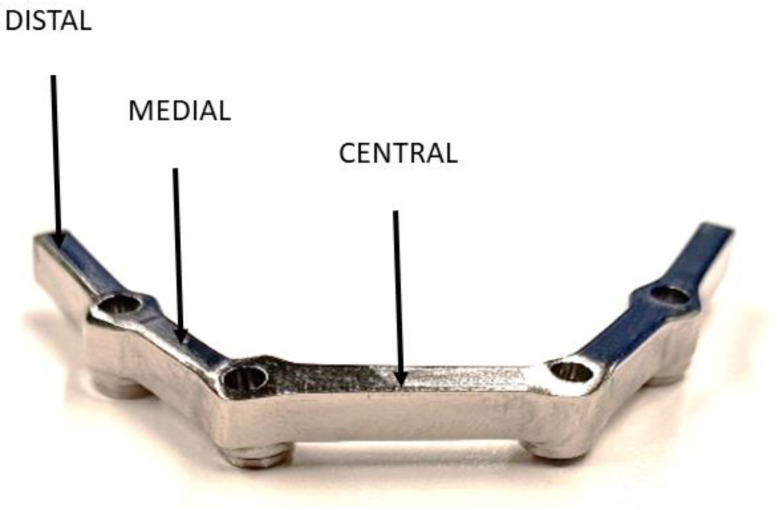
Prosthetic structure obtained for the mechanical tests.

For the validation of the results obtained in the simulation by the finite element method, analyzed structures with the same dimensions and geometries have been elaborated with the three materials studied. Five samples have been elaborated for each of the materials and for each of the positions where the mechanical stress will be exerted (distal, medial, and central). Consequently, 45 structures were elaborated. Figure 3 shows one of the structures obtained.

**Figure 4:**
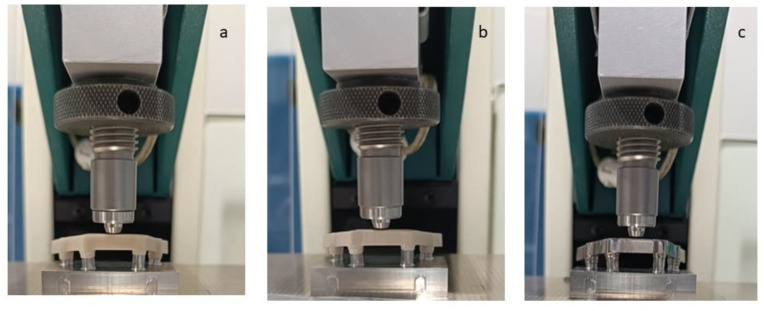
Flexural mechanical test (**a**) G-PMMA, (**b**) PEEK, (**c**) Ti6Al4V.

The mechanical tests were performed using an MTS-Bionix servo-hydraulic testing machine (Bionix 358, MTS, Minneapolis, MN, USA). The tests performed are single-point bending tests, the same as those performed in the simulation. The load application rate was 5 N/s. Figure 4 shows specimens of the three materials prepared for testing. Five mechanical tests were performed for each material and each loading position. All results were expressed as mean and standard deviation except for the bacterial adhesion test results which were expressed as median and standard error. The comparative *t*-test (with the Excel software) was carried out between the different groups at 95%, which means that for values of *p* < 0.05, there are significant differences.

## 3. Results

This section presents the results obtained for all the models of each different load condition studied. The results are divided into subsections. Each subsection includes all the load conditions of a material while a final subsection presents graphs that summarize all the results obtained.

### 3.1. Results of the Model Made of G-PMMA for the Different Load Conditions Under Study

**Figure 5:**
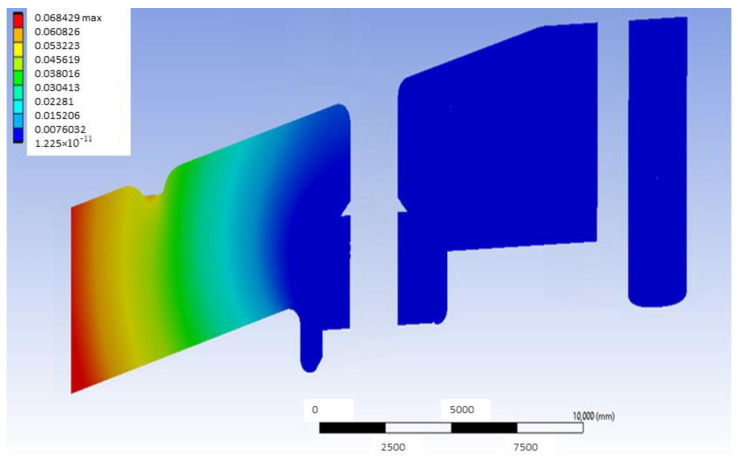
Mid-plane deformation map of the distally loaded G-PMMA model (units: mm).

**Figure 6:**
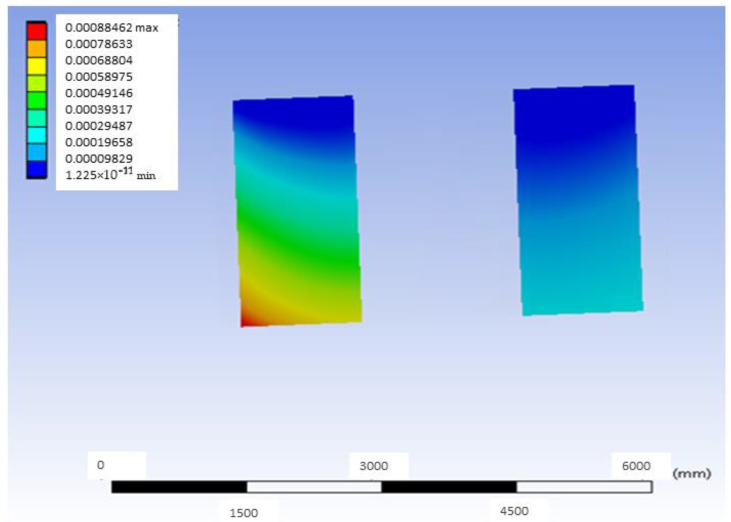
Deformation map of the Ti6Al4V bushing most affected by the application of the distal load in the G-PMMA model (units: mm).

**Figure 7:**
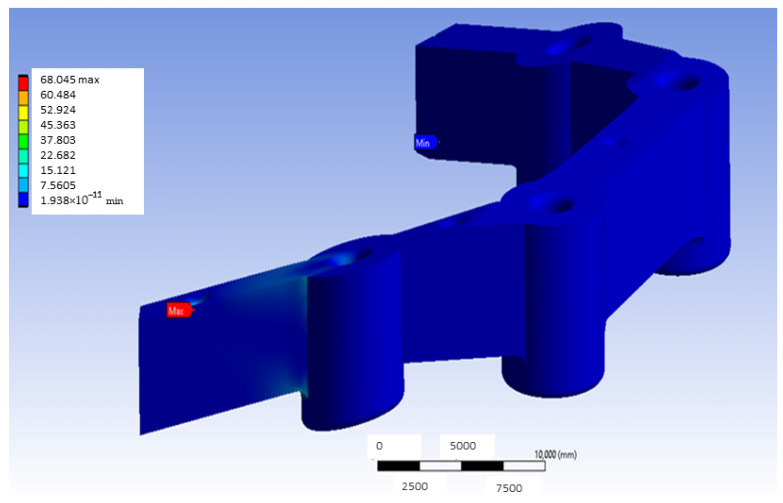
Stress map of the G-PMMA model with distal loading (units: MPa).

Figure 5 shows the deformation map in a mid-plane of the model that uses G-PMMA as a base material and has inserts of Ti6Al4V and presents the load located in the distal position. This median plane is obtained by joining the centers of the point of application of the load and the axis of the inserts. As can be observed, the largest deformations are shown in the area closer to the point where the load is applied. In the same way, Figure 6 shows the deformation map corresponding to the bushing of this model with the same load condition, most affected by the application of the load, and Figure 7 shows the stress map of the G-PMMA model with distal loading.

As can be seen in Figure 7, the maximum stress value obtained is 68.045 MPa. This maximum value is close to 65 MPa, which is the elastic limit of G-PMMA. Please note that this figure determined the magnitude of the force applied in all cases. Having obtained the general von Mises equivalent stress map, we proceeded to study the most loaded zone of the experiment, in other words, the area of the inserts. Furthermore, it is necessary to point out that, although slices of the objects are shown by the plane indicated before to facilitate visualization, this plane does not necessarily contain the maximum equivalent stress, even if it contains the surface of application of the load or the embedment. This is because in complex geometries, like the one under study in the present research, it would be difficult to know a priori where the maximum load will be found and, also, in many cases, the plane in which such area is contained changes from one model to another.

**Figure 8:**
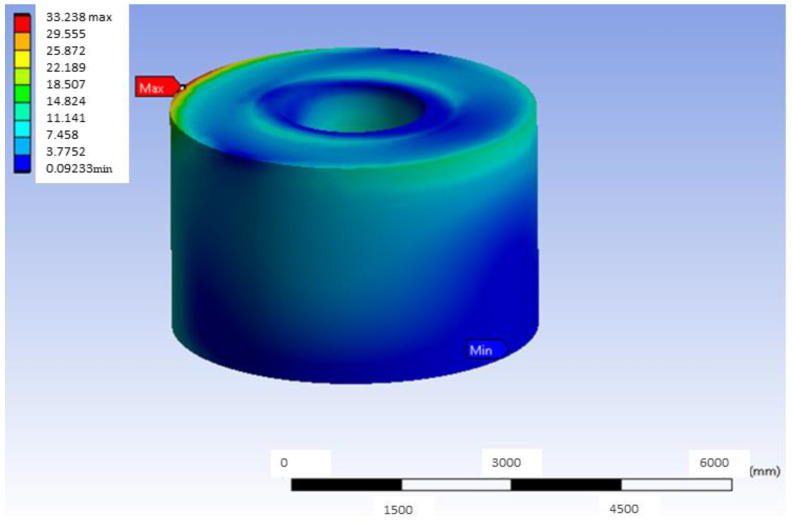
Stress map of the most loaded bushing of Ti6Al4V in the G-PMMA model with distal load.

The deformation presented in Figure 6 is complemented by Figure 8, which shows the stress map of the most loaded bushing of this model in distal load. In the same way as the figures shown before, in Figure 8, there is a concentration of stress in the upper left zone, so that in real practice, it would be expected that the model would erode or deform, and the load in that zone in real cases would be somewhat lower than that of the theoretical model. In other words, it would be possible that, in practice, in the red zone, erosion or deformation would occur and this would reduce the real stress value.

The most important results obtained for the G-PMMA model with distal load applied are shown in Table 2, which presents the global displacement (minimum and maximum), the combined von Mises stress (minimum and maximum), and the maximum strain of the assembly that forms Model 1, while the data for the area of the inserts are presented in Table 2. Also, another table that presents the summarized results for the most loaded bushing of the same model are presented in Table 3. This scheme of results layout will be repeated throughout the nine models under study.

The same kind of analysis was performed for the G-PMMA models with loads in medial and central positions. In order to keep the present scientific article within a reasonable length, no figures of either of them are included in this paper, but tabulated summarized results are presented in Table 4 for the G-PMMA model with medial load and in Table 5 for its most loaded bushing, while Table 6 presents global results for the G-PMMA model with distal load, and Table 7 details the results of the most loaded bushing of the same model.

### 3.2. Results of the Model Made of PEEK for the Different Load Conditions Under Study

**Figure 9:**
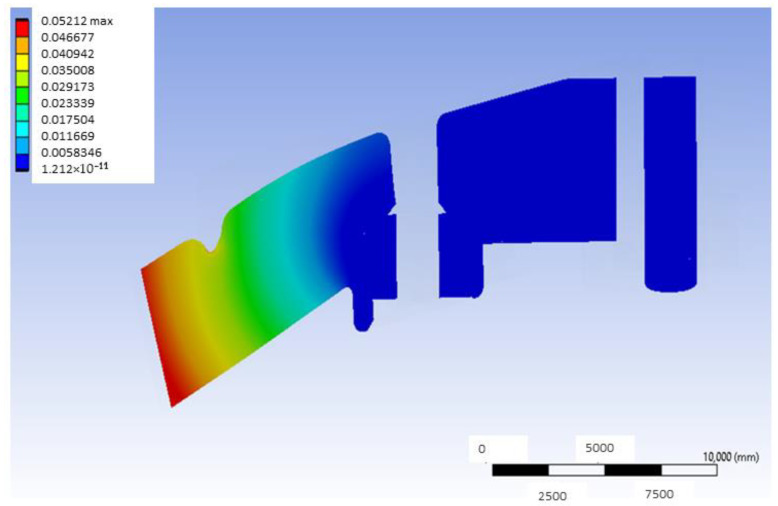
Mid-plane deformation map of the distally loaded PEEK model (units: mm).

The methodology employed for the analysis of the model made of PEEK was the same as the G-PMMA model. In this section, equivalent figures to those presented for G-PMMA in Section 3.1 are presented. Figure 9 shows the deformation map in a mid-plane of the PEEK model with Ti6Al4V inserts. This figure presents the load located in the distal position. The median plane was obtained with the same methodology as in the G-PMMA model. As can be seen in the referred figure, the deformation map in the plane is continuous, a fact that validates that the geometrical modeling has been correct since a smooth transition of the load between components is achieved. This fact is present in all the models under analysis in this research. It is important to consider that the load is applied vertically downwards, and that the model is embedded in the inserts, so that a state of triaxial stresses with a certain buckling component is produced.

**Figure 10:**
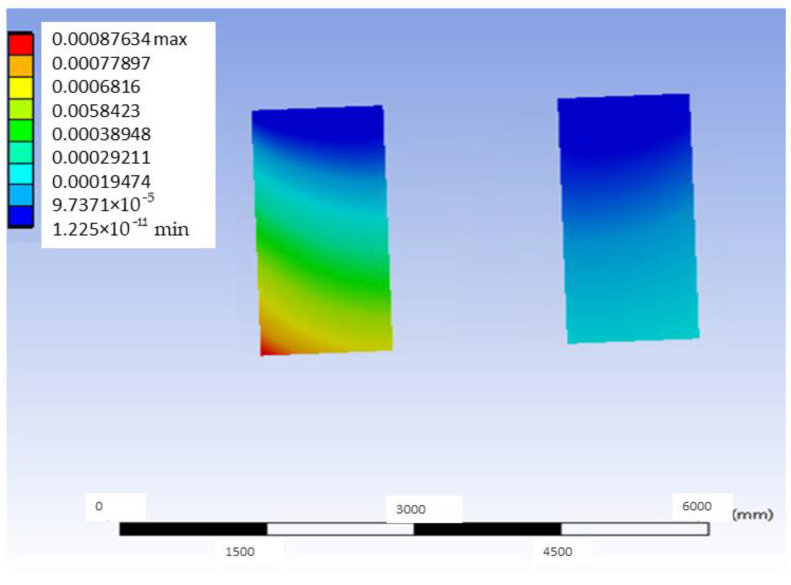
Deformation map of the Ti6Al4V bushing most affected by the application of the distal load in the PEEK model (units: mm).

**Figure 11:**
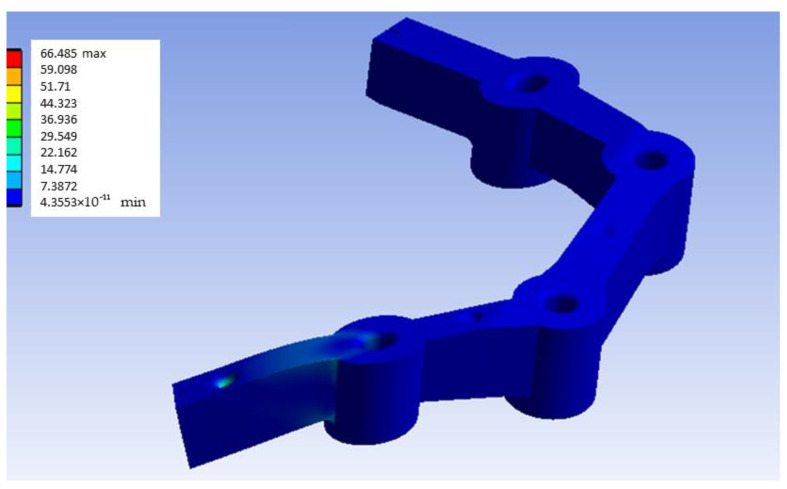
Stress map of the PEEK model with distal loading (units: MPa).

Figure 10 shows the deformation map corresponding to the bushing of the PEEK material model with distal load most affected by the application of the referred load. Also, Figure 11 shows the stress map of the PEEK model with distal loading.

**Figure 12:**
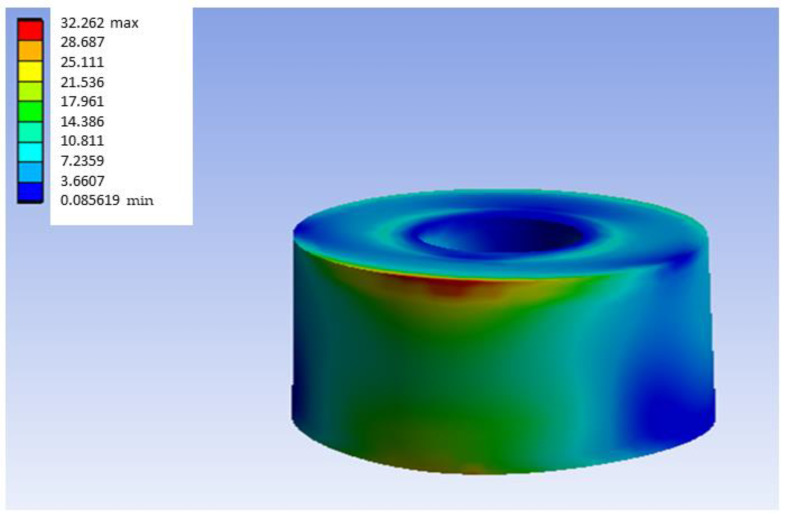
Stress map of the most loaded bushing of Ti6Al4V in the PEEK model with distal load.

As can be seen in Figure 11, the maximum stress value obtained in the PEEK model with distal loading is 66.485 MPa. Having obtained the general von Mises equivalent stress map, the area of the inserts is considered the most critical for the present experiment; the graphical results of the most loaded bushing are presented in Figure 12. As with the G-PMMA model, there is a concentration of stress in the upper left zone, so that in real practice it would be expected that the model would erode or deform, and the load in that zone would be somewhat lower than that of theoretical model. In other words, it would be possible that in the red zone, erosion or deformation would occur and this would reduce the real stress.

As in the case of the G-PMMA material, for the PEEK models with the load in medial and central positions, studies equivalent to those carried out with the model with the load in the distal position were performed; therefore, analogous tables are presented. Table 8 presents a summary of the results obtained for PEEK model with distal load while Table 9 presents the results of the most loaded bushing in the same model.

In the following tables, equivalent results to those presented for PEEK models with medial and central loads are detailed. Thus, following the same system, Table 10 summarizes the results obtained for the PEEK model with medial load, while Table 11 shows the results obtained for the most loaded bushing in this model. In the same way, Table 12 and Table 13 present the results obtained for the PEEK model with central load and the results of the most loaded bushing of such model, respectively.

### 3.3. Results of the Model Made of Ti6Al4V for the Different Load Conditions Under Study

**Figure 13:**
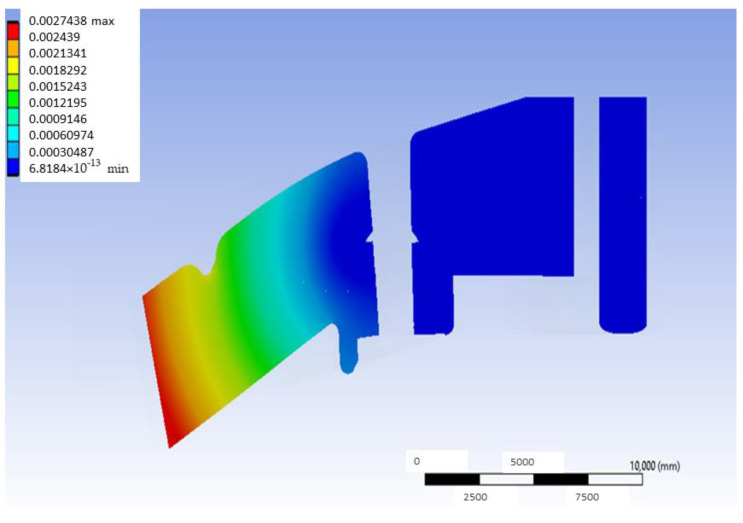
Mid-plane deformation map of the distally loaded Ti6Al4V model (units: mm).

**Figure 14:**
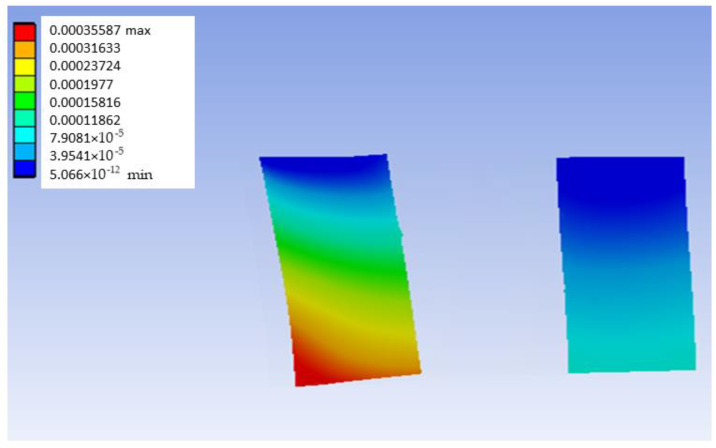
Deformation map of the Ti6Al4V bushing most affected by the application of the distal load in the Ti6Al4V model (units: mm).

The methodology employed for the analysis of the model made of Ti6Al4V was the same as for the other two materials. Figure 13 shows the deformation map in the mid-plane of the Ti6Al4V model with distal load. This figure presents the load located in the distal position. This figure is of special relevance, given that continuity can be observed between the area of the bushing, and the rest of the model, which, in this case, is made of the same material, so continuity was expected. The figure serves to verify compliance with the theory and the proper functioning of the model’s contacts. Figure 14 shows the deformation map corresponding to the bushing most affected by the application of the load.

**Figure 15:**
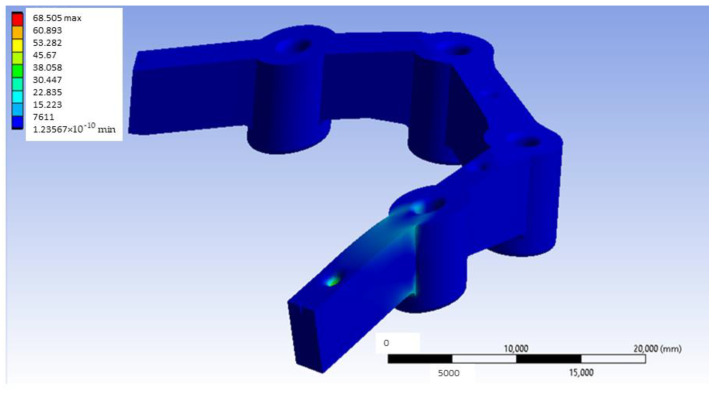
Stress map of the Ti6Al4V model with distal loading (units: MPa).

**Figure 16:**
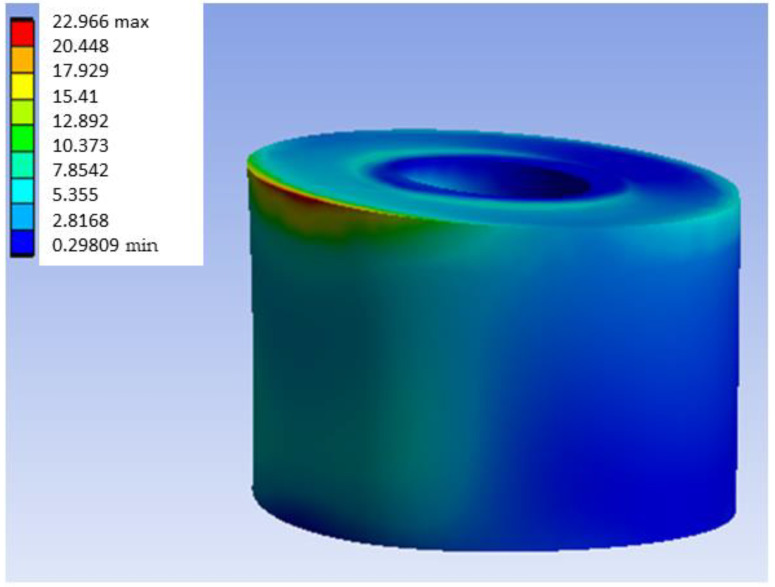
Stress map of the most loaded bushing of Ti6Al4V in the Ti6Al4V model with distal load.

Figure 15 shows the stress map of the Ti6Al4V model with distal loading. As is presented in Figure 15, the maximum stress obtained was 68.505 MPa. After generating the von Mises equivalent stress map, we focus on examining the most critical area relevant to the experiment’s practical application: the region surrounding the inserts. More specifically, Figure 16 presents the most loaded bushing of the Ti6Al4V model with distal loading.

As with the other materials, there is a concentration of stress in the upper left zone. For the case of the Ti6Al4V models, the equivalent tables for the same load conditions as in the two previous materials will be presented. Table 14 contains a summary of the results obtained for the distal load Ti6Al4V model, while Table 15 presents the results obtained for the most loaded bushing of such model. Table 16 summarizes of results obtained for medial load Ti6Al4V model. Table 17 shows the results obtained for the most loaded bushing in the medial load TiAlV4 model. Table 18 and Table 19 show the results obtained for central load and the results obtained for the most loaded bushing in the central load Ti6Al4V model, respectively.

### 3.4. Comparison of the Results Obtained for the Models and Load Conditions Under Study

In this section and as a summary, a series of graphs are presented with the maximum values of stresses and deformations both in the set of models under analysis and in the bushings that support the higher stresses values in each of the models.

**Figure 17:**
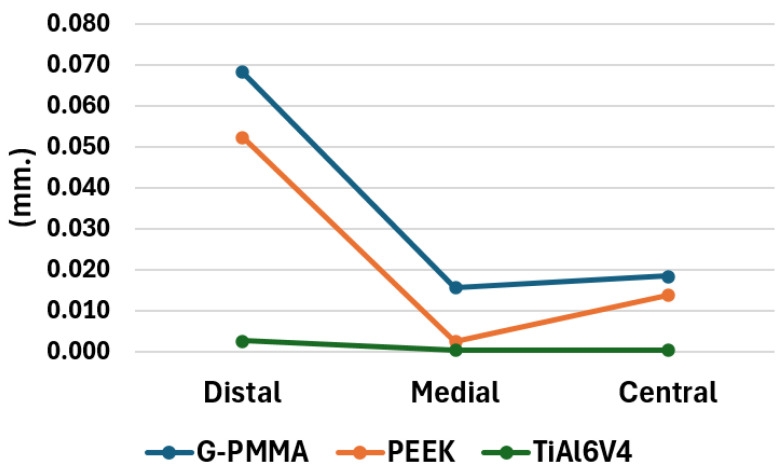
Maximum deformation of all the models (in mm.) by materials and force position.

The maximum deformation values in the three models for the loading conditions under study are presented in Figure 17. As can be observed, in all the materials analyzed, the highest deformation values correspond to the load applied in the distal position, followed by the applied in a central position and, finally, by the load applied in a medial position. Furthermore, the largest deformations on average for the three load conditions correspond to the models made of G-PMMA, followed by the PEEK models and, finally, by those made entirely of Ti6Al4V. With regard to the order of the materials, it can be said that it was the expected one, given that it corresponds to the same order as their Young’s modulus values, from lowest to highest. Also, with regard to the magnitude of the maximum deformation of the assembly versus the place of load application, it is also consistent that the position that produces the greatest deformations is the distal one, given that a lever arm is created in the structure that leads to higher displacement values induced by the load.

**Figure 18:**
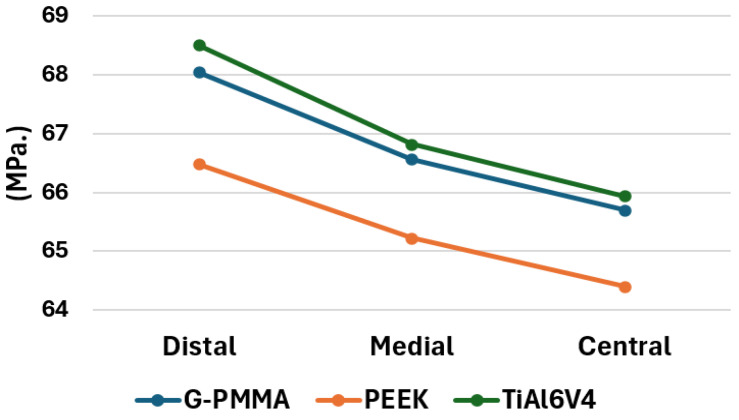
Maximum stress values according to the von Mises criteria of all the models (MPa.) by materials and force position.

The comparison of the maximum stress values achieved according to the von Mises criteria in the models of the three materials and for the three loading conditions under study are presented in Figure 18. Taking into account what is shown in the referred Figure, it is possible to affirm that, in all cases, the maximum stress value occurs for the models with loads applied in the distal position, followed by the models with load in the medial position and, finally, the models with load in the central position. Furthermore, with the same position of the load, in all cases, the highest stress values are reached in those models that are made entirely of Ti6Al4V, followed by the models made of G-PMMA, except for its bushings and, finally, the PEEK models. That is, the inverse order to that of the deformations and which means that those models in which greater displacements occur generally reach lower values of maximum stresses.

**Figure 19:**
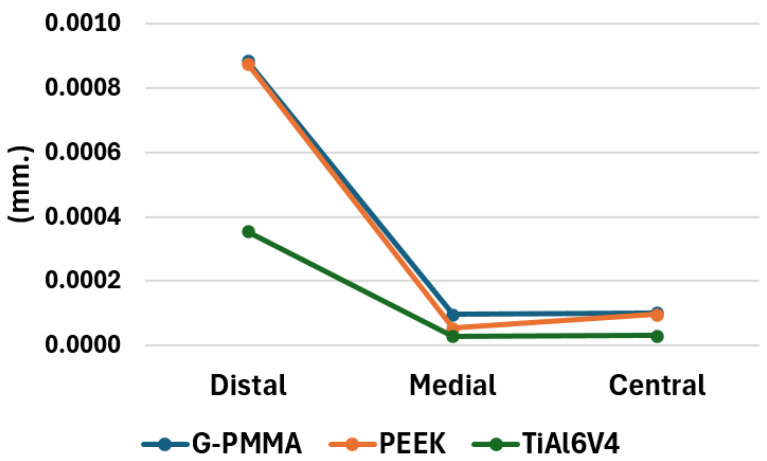
Maximum deformation values (in mm.) achieved in the most loaded bushes of each model by materials and force position.

Figure 19 presents the maximum deformation values reached in the most loaded bushings of all the models under study. As can be seen in the referred Figure, the deformations of the most loaded Ti6Al4V bushings in each of the models under study follow a similar trend, but the greatest deformations correspond to those loaded in the distal position, followed by the central and medial positions. Also, the bushings that present the greatest deformation are those corresponding to the models made of G-PMMA and PEEK, with very similar results. Although the deformation values in the medial and central position are similar, those in the medial position are somewhat higher. In this case, the highest strain values are also reached for the G-PMMA model, followed by the PEEK and, finally, the Ti6Al4V. Regarding the models with loading in the central position, almost indistinguishably, there are the bushings corresponding to the G-PMMA and PEEK models, while the maximum strain in the Ti6Al4V model is somewhat lower.

**Figure 20:**
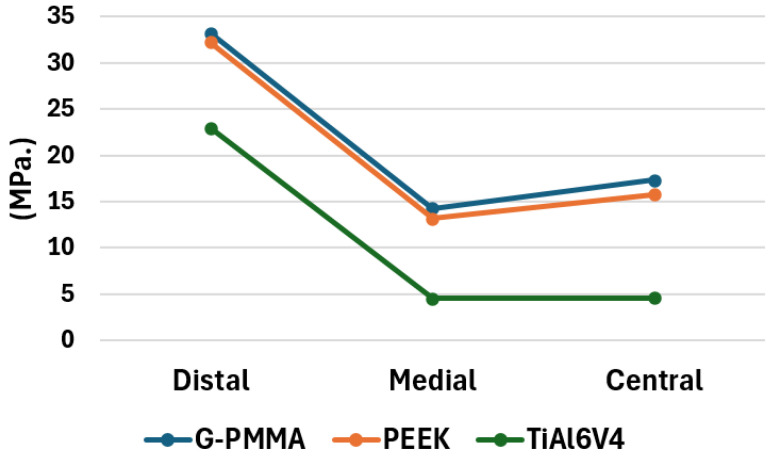
Maximum stress by von Mises criteria (in MPa) achieved in the most loaded bushes of each model by materials and force position.

**Figure 21:**
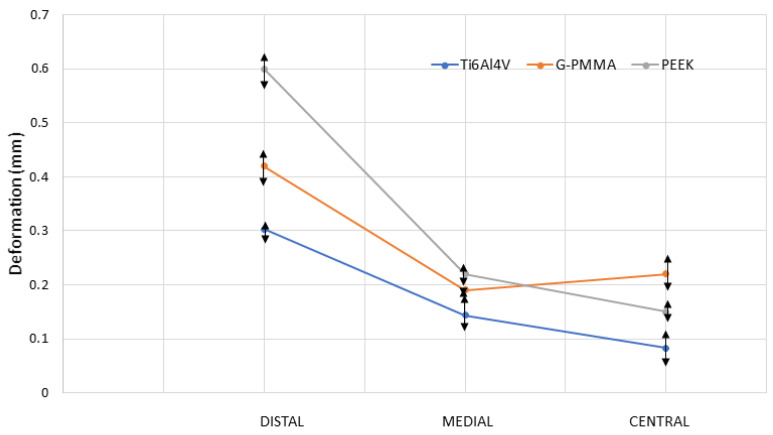
Deformation in millimeters obtained under a load of 150 N in flexural tests.

Figure 20 presents the maximum von Mises stresses reached in the most loaded bushings of each model, which, in all cases, are made of Ti6Al4V. As can be observed in this figure, the highest values correspond to the models to which load has been applied in the distal position, followed by those with the load in medial position and, finally, those with the load in a central position. Regarding the materials of the different models, the highest values in all cases correspond to models made of G-PMMA, followed by slightly lower values for those models of PEEK, while the maximum deformation values of the bushing inserted in the Ti6Al4V models are noticeably lower. Figure 21 shows the values of the mechanical tests performed and the determination of the displacement in millimeters of the sample. Displacement values were obtained when the force applied in each of the positions was 150 N. As can be seen from the results, the distal position is the one that offers the greatest deformation, as in the case of the simulation that can be seen in Figure 17 and Figure 19. The displacements decrease sharply for the medial and central positions, with the Ti6Al4V material offering the lowest displacement values in all the positions studied. The structures made of G-PMMA and PEEK show similar values although PEEK offers higher displacement in the distal position equaling the values in the medial position and slightly increases the displacement in the central position for the G-PMMA. From the results obtained, it can be said that the mechanical tests reproduce the behavior simulated by the finite element method both in the positions of application of the mechanical load on the structure and in the type of material studied in the fabrication of the prosthetic structures.

## 4. Discussion

One of the strengths of the present research is that all the models analyzed share the same mesh and geometry which makes the comparison among materials and load conditions easy. That is because the discrepancies observed among models will arise solely from variations in the materials and the different applied loading conditions. When speaking about different materials models, the one with the most different behavior when compared with other materials is Ti6Al4V [43].

The general behavior of G-PMMA and PEEK in the test performed is similar. About maximum deformations in the model, it has been found that deformations are higher in the G-PMMA models when compared to those made of PEEK. This result was expected, primarily due to differences in their mechanical properties. PEEK is a high-performance thermoplastic with significantly superior stiffness, strength, and thermal stability. These characteristics enable PEEK to resist deformation under mechanical stress, particularly at elevated temperatures, making it more rigid and resistant to strain. In contrast, G-PMMA, although reinforced with graphene fibers to enhance its strength, retains the inherent limitations of a polymeric material, with a lower modulus of elasticity and tensile strength compared to PEEK. As a result, G-PMMA is more prone to deformation when subjected to mechanical loads. Therefore, the higher deformation observed in G-PMMA is a predictable outcome based on the inherent mechanical properties of these two materials [44,45,46].

The highest values of maximum stress according to the von Mises criteria are achieved in models made of Ti6Al4V, followed by G-PMMA and PEEK. These stress values are primarily governed by the distinct mechanical properties inherent to each material. Ti6Al4V, being a titanium alloy, possesses a significantly higher strength-to-weight ratio compared to the other two materials. This alloy is designed to withstand high mechanical stresses, exhibiting excellent tensile strength and resistance to fatigue and deformation. As a result, when subjected to loads, Ti6Al4V can absorb a higher amount of stress before undergoing plastic deformation, which explains why it shows the highest stress values among the three materials. Its strength is further enhanced by its metallic structure, which allows for efficient load distribution and retention of structural integrity under extreme conditions [47,48].

In contrast, G-PMMA is a polymer composite, reinforced with graphene fibers to improve its mechanical properties over pure PMMA. While the addition of graphene fibers enhances its strength, it remains a thermoplastic material with lower intrinsic stiffness and tensile strength compared to titanium alloys. As such, G-PMMA is more prone to plastic deformation under stress compared to Ti6Al4V. However, due to its relatively higher stiffness compared to other common polymers, G-PMMA is able to withstand moderate stress levels before significant deformation occurs, placing it in the intermediate position between Ti6Al4V and PEEK in terms of stress capacity. Lastly, PEEK, although a high-performance thermoplastic with excellent mechanical properties, including high strength and thermal stability, is still a polymer material and thus inherently more susceptible to deformation under high loads compared to metals. While PEEK is known for its excellent chemical resistance, toughness, and dimensional stability, its intrinsic modulus of elasticity and tensile strength are lower than those of both Ti6Al4V and G-PMMA. Consequently, PEEK experiences higher deformation under applied loads and reaches a lower stress value before yielding. Its behavior under load is governed by the molecular structure of the polymer, which, although optimized for performance, cannot match the superior stress resistance provided by metallic materials such as Ti6Al4V.

In the case of the maximum von Mises stress and deformation values achieved in the most loaded bushing of each model, values of G-PMMA and PEEK models are really close and in some cases they would even be considered within the 0.2% of the uncertainty confidence interval of error due to the model convergence. Please note that in all models, bushes are made of Ti6Al4V alloy.

Also, it should be noted that there is also a difference in the results obtained depending on the applied load, whether distal, medial, or central, showing that in all simulations, it is the distal test that offers the worst results in terms of presenting a higher value for both displacement and tension. This situation would be mitigated if the prosthesis cantilever is reduced. Likewise, it is also advisable to check whether the base model in PEEK or G-PMMA with Ti6Al4V bushings can afford a displacement map 10 times greater (an order of magnitude) than that obtained with the model made entirely of Ti6Al4V. Therefore, the further the loading point is from a strong bond, the more global displacement there is, and this is proportional to the unitary displacement, which is proportional to Young’s modulus, which depends on the material, which is why what we see happens. The displacements in the bushings are similar, because Ti6Al4V is much more rigid than the other two materials. Overall, the graph suggests that the distal loading position should be studied in order to find if it can be restricted in some way so that the stress does not generate as much deformation compared to the rest.

The present study is subject to certain limitations inherent to finite element analyses in biomechanics. A key simplifying assumption is the use of linear-elastic material models for all the pieces involved in these models. In fact, materials have been considered homogeneous and isotropic. While these assumptions facilitate computational efficiency and are widely used in similar studies, they do not account for the non-linear and time-dependent behavior of biological tissues, such as viscoelasticity or plastic deformation under high loads.

The use of linear models, simplifying the contacts between elements, can also partially distort the results, an effect that we have tried to minimize using loads that ensure that the material works below the elastic limit at all conditions. Also, the geometries used, faithful reproductions of the real models, generate stress concentrations, which in tests and simulations by the finite element method often do not occur because the geometry used has been simplified (the fact being that maintaining visual verisimilitude has hardly been practiced in these models). This generates load values higher than the real ones, concentrated in the “corners” and in other singularities, which, in a laboratory test, would disappear practically immediately since the physical object would suffer from erosion or deformation.

Boundary conditions were also idealized to represent a controlled in silico environment with fully fixed supports, although these idealizations may not fully replicate the complex mechanical interactions occurring in vivo. Additionally, this analysis does not incorporate critical biological factors such as bone adaptation, remodeling, or degradation over time, which can significantly influence the long-term performance of dental prostheses. The effects of wear and fatigue, which are known to play a pivotal role in the lifespan of dental materials under repetitive loading, were also excluded.

From a clinical point of view, bruxing patients with high mechanical loads should be advised to use Ti6Al4V frameworks as polymeric frameworks may have limitations in mechanical properties. In patients with low to moderate load levels where dental esthetics is also an important part of the treatment, the polymeric materials studied can be used. In the latter case, the clinician should evaluate the type of structure and assess whether there are stresses in the distal positions with stresses with a large cantilever. In these cases, polymeric materials would not be advisable, as has been demonstrated in the mechanical tests carried out and confirmed by the simulation of the different structures by means of finite elements [36,48,49,50].

Despite these limitations, the study provides valuable insights into the biomechanical behavior of the prosthetic system under different loading conditions. Future research could integrate more advanced material models, non-linear contact interactions, and simulations of biological processes to enhance the clinical relevance of the findings.

## 5. Conclusions

Regarding the mechanical response of the materials, it was observed that the PEEK material presented a superior capacity to support high magnitude loads without compromising the structural integrity of the prosthesis. On the other hand, the G-PMMA material showed adequate resistance to deformation under moderate loads, but its performance decreased in high load scenarios, suggesting that its use is more indicated for prostheses in areas with less exposure to extreme masticatory forces. Finally, the PEEK material showed intermediate behavior in the three loading conditions, balancing strength, and flexibility, suggesting a potential for versatile use in prosthetic applications. However, it was noted that this material may require additional adjustments to maintain long-term stability in patients with variable masticatory loads. The validation of the simulation by the finite element method was carried out by means of mechanical tests and the validity of the simulation was verified. It is possible to observe that in the application of 150N of the load, the distal position is the one with the highest deformation, with a displacement of 0.6 mm for PEEK, 0.43 mm for G-PMMA, and the lowest for Ti6Al4V, which corresponds to 0.30 mm. The deformations decrease sharply for the medial and central positions decreasing to values close to 0.1 mm. G-PMMA presents the highest deformation values (0.21 mm) in the central position. The choice of the appropriate material should consider not only the strength of the material under various loading conditions, but also the adaptability of the material to the specific needs of the patient. The findings of this study provide valuable information for decision making in the selection of prosthetic materials and establish a basis for future research that can further optimize the design of dental prostheses based on realistic conditions of use.

## Figures and Tables

**Table 1:** Properties of the materials employed in the present study [18,19,22,23,24,26]. G-PMMA: glycol-modified polymethyl methacrylate (0.25% of graphene), PEEK: polyether ether ketone Ti6Al4V: titanium alloy ASTM grade V.

Material	Property	Value	Units
G-PMMA	Density	1190	kg/m^3^
	Young’s modulus	3.2 · 10^9^	N/m^2^ (Pascal)
	Poisson’s coefficient	0.35	
	Tensile yield strength	6.5 · 10^7^	Pascales
PEEK	Density	1310	kg/m^3^
	Young’s modulus	4.2 · 10^9^	N/m^2^ (Pascal)
	Poisson’s coefficient	0.38	
	Tensile yield strength	1.16 · 10^8^	Pascal
Ti6Al4V	Density	4430	kg/m^3^
	Young’s modulus	1.138 · 10^11^	N/m^2^ (Pascal)
	Poisson’s coefficient	0.342	
	Tensile yield strength	8.8·10^8^	Pascal

**Table 2:** Summary of results obtained for distal load G-PMMA model, being Def. min: Minimum deformation value in mm, Def. max: maximum deformation value in mm, VM min: minimum von Mises stress value in MPa, VM max: maximum von Mises stress value in MPa, Strain max: maximum strain value.

Force	Def. Min.	Def. Max.	VM Min.	VM Max.	Strain Max.
5 N	$2.45\cdot 10^{-12}$	$1.37\cdot 10^{-2}$	$3.88\cdot 10^{-12}$	13.609	$4.27\cdot 10^{-3}$
10 N	$4.90\cdot 10^{-12}$	$2.74\cdot 10^{-2}$	$7.75\cdot 10^{-12}$	27.218	$8.55\cdot 10^{-3}$
15 N	$7.35\cdot 10^{-12}$	$4.11\cdot 10^{-2}$	$1.16\cdot 10^{-11}$	40.827	$1.28\cdot 10^{-2}$
20 N	$9.80\cdot 10^{-12}$	$5.47\cdot 10^{-2}$	$1.55\cdot 10^{-11}$	54.436	$1.71\cdot 10^{-2}$
25 N	$1.23\cdot 10^{-11}$	$8.85\cdot 10^{-2}$	$1.94\cdot 10^{-11}$	68.045	$2.13\cdot 10^{-2}$

**Table 3:** Results obtained for the most loaded bushing in the distal load G-PMMA model, being Def. min: Minimum deformation value in mm, Def. max: maximum deformation value in mm, VM min: minimum von Mises stress value in MPa, VM max: maximum von Mises stress value in MPa.

Force	Def. Min.	Def. Max.	VM Min.	VM Max.
5 N	$2.45\cdot 10^{-12}$	$1.77\cdot 10^{-4}$	$5.39\cdot 10^{-8}$	6.6476
10 N	$4.90\cdot 10^{-12}$	$3.54\cdot 10^{-4}$	$1.08\cdot 10^{-7}$	13.295
15 N	$7.35\cdot 10^{-12}$	$5.31\cdot 10^{-4}$	$1.62\cdot 10^{-7}$	19.943
20 N	$9.80\cdot 10^{-12}$	$7.08\cdot 10^{-4}$	$2.15\cdot 10^{-7}$	26.59
25 N	$1.23\cdot 10^{-11}$	$8.85\cdot 10^{-4}$	$2.69\cdot 10^{-7}$	33.238

**Table 4:** Summary of results obtained for medial load G-PMMA model, being Def. min: Minimum deformation value in mm, Def. max: maximum deformation value in mm, VM min: minimum von Mises stress value in MPa, VM max: maximum von Mises stress value in MPa, Strain max: maximum strain value.

Force	Def. Min.	Def. Max.	VM Min.	VM Max.	Strain Max.
5 N	$3.10\cdot 10^{-25}$	$3.14\cdot 10^{-3}$	$3.85\cdot 10^{-11}$	13,314	$4.17\cdot 10^{-3}$
10 N	$6.20\cdot 10^{-25}$	$6.29\cdot 10^{-3}$	$7.70\cdot 10^{-11}$	26,628	$8.35\cdot 10^{-3}$
15 N	$9.30\cdot 10^{-25}$	$9.43\cdot 10^{-3}$	$1.16\cdot 10^{-10}$	39,942	$1.25\cdot 10^{-2}$
20 N	$1.24\cdot 10^{-24}$	$1.26\cdot 10^{-2}$	$1.54\cdot 10^{-10}$	53,256	$1.67\cdot 10^{-2}$
25 N	$1.55\cdot 10^{-24}$	$1.57\cdot 10^{-2}$	$1.93\cdot 10^{-10}$	66,570	$2.09\cdot 10^{-2}$

**Table 5:** Results obtained for the most loaded bushing in the medial load G-PMMA model, being Def. min: Minimum deformation value in mm, Def. max: maximum deformation value in mm, VM min: minimum von Mises stress value in MPa, VM max: maximum von Mises stress value in MPa.

Force	Def. Min.	Def. Max.	VM Min.	VM Max.
5 N	$3.10\cdot 10^{-25}$	$1.93\cdot 10^{-5}$	$6.90\cdot 10^{-7}$	2.8598
10 N	$6.20\cdot 10^{-25}$	$3.85\cdot 10^{-5}$	$1.38\cdot 10^{-6}$	5.7196
15 N	$9.30\cdot 10^{-25}$	$5.77\cdot 10^{-5}$	$2.07\cdot 10^{-6}$	8.5794
20 N	$1.24\cdot 10^{-24}$	$7.70\cdot 10^{-5}$	$2.76\cdot 10^{-6}$	11.439
25 N	$1.55\cdot 10^{-24}$	$9.62\cdot 10^{-5}$	$3.45\cdot 10^{-6}$	14.299

**Table 6:** Summary of results obtained for central load G-PMMA model, being Def. min: Minimum deformation value in mm, Def. max: maximum deformation value in mm, VM min: minimum von Mises stress value in MPa, VM max: maximum von Mises stress value in MPa, Strain max: maximum strain value.

Force	Def. Min.	Def. Max.	VM Min.	VM Max.	Strain Max.
5 N	0	$3.72\cdot 10^{-3}$	$2.87\cdot 10^{-9}$	13,140	$4.12\cdot 10^{-3}$
10 N	0	$7.43\cdot 10^{-3}$	$5.74\cdot 10^{-9}$	26,280	$8.25\cdot 10^{-3}$
15 N	0	$1.11\cdot 10^{-2}$	$8.60\cdot 10^{-9}$	39,421	$1.24\cdot 10^{-2}$
20 N	0	$1.49\cdot 10^{-2}$	$1.15\cdot 10^{-8}$	52,561	$1.65\cdot 10^{-2}$
25 N	0	$1.86\cdot 10^{-2}$	$1.43\cdot 10^{-8}$	65,701	$2.06\cdot 10^{-2}$

**Table 7:** Results obtained for the most loaded bushing in the central load G-PMMA model, being Def. min: Minimum deformation value in mm, Def. max: maximum deformation value in mm, VM min: minimum von Mises stress value in MPa, VM max: maximum von Mises stress value in MPa.

Force	Def. Min.	Def. Max.	VM Min.	VM Max.
5 N	0	$2.03\cdot 10^{-5}$	$5.14\cdot 10^{-5}$	3.4705
10 N	0	$4.05\cdot 10^{-5}$	$1.03\cdot 10^{-4}$	6.9409
15 N	0	$6.08\cdot 10^{-5}$	$1.54\cdot 10^{-4}$	10.411
20 N	0	$8.11\cdot 10^{-5}$	$2.06\cdot 10^{-4}$	13.882
25 N	0	$1.01\cdot 10^{-4}$	$2.57\cdot 10^{-4}$	17.352

**Table 8:** Summary of results obtained for distal load PEEK model, being Def. min: Minimum deformation value in mm, Def. max: maximum deformation value in mm, VM min: minimum von Mises stress value in MPa, VM max: maximum von Mises stress value in MPa, Strain max: maximum strain value.

Force	Def. Min.	Def. Max.	VM Min.	VM Max.	Strain Max.
5 N	$2.43\cdot 10^{-12}$	$1.05\cdot 10^{-2}$	$8.72\cdot 10^{-12}$	13.297	$3.18\cdot 10^{-3}$
10 N	$4.85\cdot 10^{-12}$	$2.10\cdot 10^{-2}$	$1.74\cdot 10^{-11}$	26.594	$6.36\cdot 10^{-3}$
15 N	$7.28\cdot 10^{-12}$	$3.15\cdot 10^{-2}$	$2.61\cdot 10^{-11}$	39.891	$9.55\cdot 10^{-3}$
20 N	$9.70\cdot 10^{-12}$	$4.20\cdot 10^{-2}$	$3.48\cdot 10^{-11}$	53.188	$1.27\cdot 10^{-2}$
25 N	$1.21\cdot 10^{-11}$	$5.25\cdot 10^{-2}$	$4.36\cdot 10^{-11}$	66.485	$1.59\cdot 10^{-2}$

**Table 9:** Results obtained for the most loaded bushing in the distal load PEEK model, being Def. min: Minimum deformation value in mm, Def. max: maximum deformation value in mm, VM min: minimum von Mises stress value in MPa, VM max: maximum von Mises stress value in MPa.

Force	Def. Min.	Def. Max.	VM Min.	VM Max.
5 N	$2.43\cdot 10^{-12}$	$1.75\cdot 10^{-4}$	$9.26\cdot 10^{-8}$	6.4523
10 N	$4.85\cdot 10^{-12}$	$3.51\cdot 10^{-4}$	$1.85\cdot 10^{-7}$	12.905
15 N	$7.28\cdot 10^{-12}$	$5.26\cdot 10^{-4}$	$2.78\cdot 10^{-7}$	19.357
20 N	$9.70\cdot 10^{-12}$	$7.01\cdot 10^{-4}$	$3.70\cdot 10^{-7}$	25.809
25 N	$1.21\cdot 10^{-11}$	$8.76\cdot 10^{-4}$	$4.63\cdot 10^{-7}$	32.262

**Table 10:** Summary of results obtained for medial load PEEK model, being Def. min: Minimum deformation value in mm, Def. max: maximum deformation value in mm, VM min: minimum von Mises stress value in MPa, VM max: maximum von Mises stress value in MPa, Strain max: maximum strain value.

Force	Def. Min.	Def. Max.	VM Min.	VM Max.	Strain Max.
5 N	$2.67\cdot 10^{-13}$	$5.15\cdot 10^{-4}$	$4.09\cdot 10^{-11}$	13.045	$3.12\cdot 10^{-3}$
10 N	$5.34\cdot 10^{-13}$	$1.03\cdot 10^{-3}$	$8.18\cdot 10^{-11}$	26.090	$6.23\cdot 10^{-3}$
15 N	$8.01\cdot 10^{-13}$	$1.55\cdot 10^{-3}$	$1.23\cdot 10^{-10}$	39.135	$9.35\cdot 10^{-3}$
20 N	$1.07\cdot 10^{-12}$	$2.06\cdot 10^{-3}$	$1.64\cdot 10^{-10}$	52.180	$1.25\cdot 10^{-2}$
25 N	$1.33\cdot 10^{-12}$	$2.58\cdot 10^{-3}$	$2.04\cdot 10^{-10}$	65.225	$1.56\cdot 10^{-2}$

**Table 11:** Results obtained for the most loaded bushing in the distal load PEEK model, being: Def. min: Minimum deformation value in mm, Def. max: maximum deformation value in mm, VM min: minimum von Mises stress value in MPa, VM max: maximum von Mises stress value in MPa.

Force	Def. Min.	Def. Max.	VM Min.	VM Max.
5 N	$2.67\cdot 10^{-13}$	$1.08\cdot 10^{-5}$	$8.30\cdot 10^{-7}$	2.6418
10 N	$5.34\cdot 10^{-13}$	$2.15\cdot 10^{-5}$	$1.66\cdot 10^{-6}$	5.2836
15 N	$8.01\cdot 10^{-13}$	$3.23\cdot 10^{-5}$	$2.49\cdot 10^{-6}$	7.9254
20 N	$1.07\cdot 10^{-12}$	$4.31\cdot 10^{-5}$	$3.32\cdot 10^{-6}$	10.567
25 N	$1.33\cdot 10^{-12}$	$5.39\cdot 10^{-5}$	$4.15\cdot 10^{-6}$	13.209

**Table 12:** Summary of results obtained for central load PEEK model, being: Def. min: Minimum deformation value in mm, Def. max: maximum deformation value in mm, VM min: minimum von Mises stress value in MPa, VM max: maximum von Mises stress value in MPa, Strain max: maximum strain value.

Force	Def. Min.	Def. Max.	VM Min.	VM Max.	Strain Max.
5 N	0	$2.79\cdot 10^{-3}$	$2.83\cdot 10^{-9}$	12.881	$3.08\cdot 10^{-3}$
10 N	0	$5.58\cdot 10^{-3}$	$5.65\cdot 10^{-9}$	25.761	$6.16\cdot 10^{-3}$
15 N	0	$8.37\cdot 10^{-3}$	$8.48\cdot 10^{-9}$	38.642	$9.24\cdot 10^{-3}$
20 N	0	$1.12\cdot 10^{-2}$	$1.13\cdot 10^{-8}$	51.523	$1.23\cdot 10^{-2}$
25 N	0	$1.39\cdot 10^{-2}$	$1.41\cdot 10^{-8}$	64.404	$1.54\cdot 10^{-2}$

**Table 13:** Results obtained for the most loaded bushing in the central load PEEK model, being: Def. min: Minimum deformation value in mm, Def. max: maximum deformation value in mm, VM min: minimum von Mises stress value in MPa, VM max: maximum von Mises stress value in MPa, Strain max: maximum strain value.

Force	Def. Min.	Def. Max.	VM Min.	VM Max.
5 N	0	$1.93\cdot 10^{-5}$	$4.86\cdot 10^{-5}$	3.1576
10 N	0	$3.86\cdot 10^{-5}$	$9.71\cdot 10^{-5}$	6.3152
15 N	0	$5.79\cdot 10^{-5}$	$1.46\cdot 10^{-4}$	9.4727
20 N	0	$7.71\cdot 10^{-5}$	$1.94\cdot 10^{-4}$	12.63
25 N	0	$9.64\cdot 10^{-5}$	$2.43\cdot 10^{-4}$	15.788

**Table 14:** Summary of results obtained for distal load Ti6Al4V model, being Def. min: Minimum deformation value in mm, Def. max: maximum deformation value in mm, VM min: minimum von Mises stress value in MPa, VM max: maximum von Mises stress value in MPa, Strain max: maximum strain value.

Force	Def. Min.	Def. Max.	VM Min.	VM Max.	Strain Max.
5 N	$1.36\cdot 10^{-13}$	$5.49\cdot 10^{-4}$	$2.51\cdot 10^{-11}$	13.701	$1.21\cdot 10^{-4}$
10 N	$2.73\cdot 10^{-13}$	$1.10\cdot 10^{-3}$	$5.03\cdot 10^{-11}$	27.402	$2.42\cdot 10^{-4}$
15 N	$4.09\cdot 10^{-13}$	$1.65\cdot 10^{-3}$	$7.54\cdot 10^{-11}$	41.103	$3.63\cdot 10^{-4}$
20 N	$5.45\cdot 10^{-13}$	$2.20\cdot 10^{-3}$	$1.01\cdot 10^{-10}$	54.804	$4.84\cdot 10^{-4}$
25 N	$6.82\cdot 10^{-13}$	$2.74\cdot 10^{-3}$	$1.26\cdot 10^{-10}$	68.505	$6.05\cdot 10^{-4}$

**Table 15:** Results obtained for the most loaded bushing in the distal load Ti6Al4V model, being Def. min: Minimum deformation value in mm, Def. max: maximum deformation value in mm, VM min: minimum von Mises stress value in MPa, VM max: maximum von Mises stress value in MPa.

Force	Def. Min.	Def. Max.	VM Min.	VM Max.
5 N	$1.01\cdot 10^{-12}$	$7.12\cdot 10^{-5}$	$5.09\cdot 10^{-7}$	4.5933
10 N	$2.03\cdot 10^{-12}$	$1.42\cdot 10^{-4}$	$1.02\cdot 10^{-6}$	9.1866
15 N	$3.04\cdot 10^{-12}$	$2.14\cdot 10^{-4}$	$1.53\cdot 10^{-6}$	13.78
20 N	$4.05\cdot 10^{-12}$	$2.85\cdot 10^{-4}$	$2.03\cdot 10^{-6}$	18.373
25 N	$5.07\cdot 10^{-12}$	$3.56\cdot 10^{-4}$	$2.54\cdot 10^{-6}$	22.966

**Table 16:** Summary of results obtained for medial load Ti6Al4V model, being Def. min: Minimum deformation value in mm, Def. max: maximum deformation value in mm, VM min: minimum von Mises stress value in MPa, VM max: maximum von Mises stress value in MPa, Strain max: maximum strain value.

Force	Def. Min.	Def. Max.	VM Min.	VM Max.	Strain Max.
5 N	$1.04\cdot 10^{-25}$	$9.20\cdot 10^{-5}$	$2.00\cdot 10^{-10}$	13.365	$1.18\cdot 10^{-4}$
10 N	$2.08\cdot 10^{-25}$	$1.84\cdot 10^{-4}$	$4.01\cdot 10^{-10}$	26.730	$2.36\cdot 10^{-4}$
15 N	$3.12\cdot 10^{-25}$	$2.76\cdot 10^{-4}$	$6.01\cdot 10^{-10}$	40.095	$3.53\cdot 10^{-4}$
20 N	$4.16\cdot 10^{-25}$	$3.68\cdot 10^{-4}$	$8.01\cdot 10^{-10}$	53.460	$4.71\cdot 10^{-4}$
25 N	$5.20\cdot 10^{-25}$	$4.60\cdot 10^{-4}$	$1.00\cdot 10^{-9}$	66.825	$5.89\cdot 10^{-4}$

**Table 17:** Results obtained for the most loaded bushing in the medial load TiAlV4 model, being Def. min: Minimum deformation value in mm, Def. max: maximum deformation value in mm, VM min: minimum von Mises stress value in MPa, VM max: maximum von Mises stress value in MPa.

Force	Def. Min.	Def. Max.	VM Min.	VM Max.
5 N	$1.04\cdot 10^{-25}$	$5.82\cdot 10^{-6}$	$5.17\cdot 10^{-6}$	0.90975
10 N	$2.08\cdot 10^{-25}$	$1.16\cdot 10^{-5}$	$1.03\cdot 10^{-5}$	1.8195
15 N	$3.12\cdot 10^{-25}$	$1.75\cdot 10^{-5}$	$1.55\cdot 10^{-5}$	2.7292
20 N	$4.16\cdot 10^{-25}$	$2.33\cdot 10^{-5}$	$2.07\cdot 10^{-5}$	3.639
25 N	$5.20\cdot 10^{-25}$	$2.91\cdot 10^{-5}$	$2.59\cdot 10^{-5}$	4.5487

**Table 18:** Summary of results obtained for central load Ti6Al4V model, being Def. min: Minimum deformation value in mm, Def. max: maximum deformation value in mm, VM min: minimum von Mises stress value in MPa, VM max: maximum von Mises stress value in MPa, Strain max: maximum strain value.

Force	Def. Min.	Def. Max.	VM Min.	VM Max.	Strain Max.
5 N	0	$1.11\cdot 10^{-4}$	$3.67\cdot 10^{-9}$	13,188	$1.16\cdot 10^{-4}$
10 N	0	$2.21\cdot 10^{-4}$	$7.33\cdot 10^{-9}$	26,376	$2.33\cdot 10^{-4}$
15 N	0	$3.32\cdot 10^{-4}$	$1.10\cdot 10^{-8}$	39,564	$3.49\cdot 10^{-4}$
20 N	0	$4.42\cdot 10^{-4}$	$1.47\cdot 10^{-8}$	52,752	$4.65\cdot 10^{-4}$
25 N	0	$5.53\cdot 10^{-4}$	$1.83\cdot 10^{-8}$	65,940	$5.82\cdot 10^{-4}$

**Table 19:** Results obtained for the most loaded bushing in the central load Ti6Al4V model, being Def. min: Minimum deformation value in mm, Def. max: maximum deformation value in mm, VM min: minimum von Mises stress value in MPa, VM max: maximum von Mises stress value in MPa.

Force	Def. Min.	Def. Max.	VM Min.	VM Max.
5 N	0	$6.22\cdot 10^{-6}$	$7.80\cdot 10^{-5}$	0.91538
10 N	0	$1.24\cdot 10^{-5}$	$1.56\cdot 10^{-4}$	1.8308
15 N	0	$1.87\cdot 10^{-5}$	$2.34\cdot 10^{-4}$	2.7461
20 N	0	$2.49\cdot 10^{-5}$	$3.12\cdot 10^{-4}$	3.6615
25 N	0	$3.11\cdot 10^{-5}$	$3.90\cdot 10^{-4}$	4.5769

## Data Availability

The data that support the findings of this study are available from the corresponding author upon reasonable request. Data are contained within the article.
